# Supplementary material for: 3D mesh processing using GAMer 2 to enable reaction-diffusion simulations in realistic cellular geometries
Source: PLoS Comput Biol. 2020 Apr 6;16(4):e1007756. doi: 10.1371/journal.pcbi.1007756 (PMC7162555; doi:10.1371/journal.pcbi.1007756)
Supplement: S1 Fig — (PDF) [file pcbi.1007756.s001.pdf]

3D Mesh processing using GAMer 2 to enable reaction-diffusion simulations in realistic cellular geometries

Christopher T. Lee, Justin G. Laughlin, Nils Angliviel de La Beaumelle, Rommie E. Amaro,
J. Andrew McCammon, Ravi Ramamoorthi, Michael Holst, and Padmini Rangamani

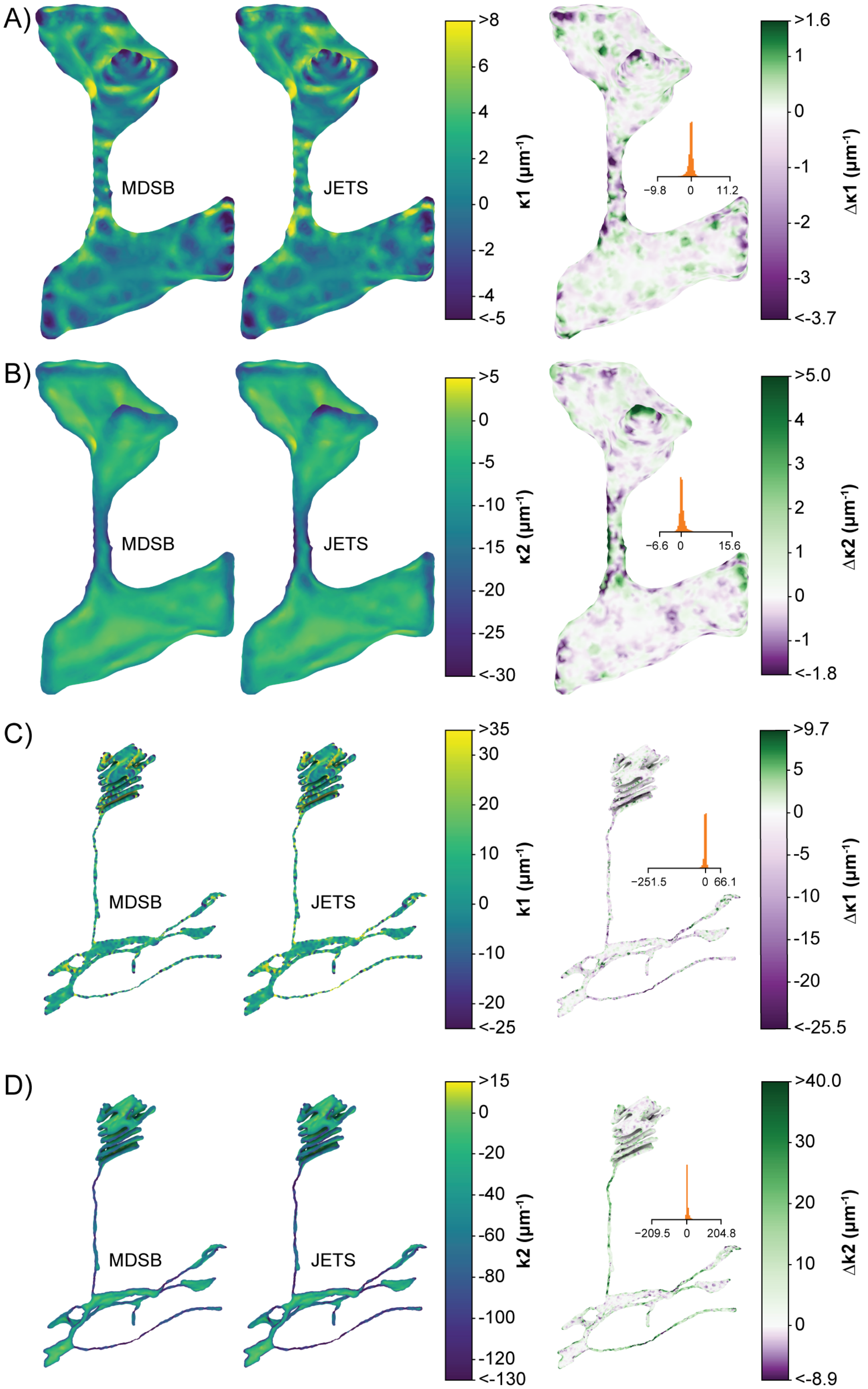
A)
MDSB
JETS
>8
6
4
2
0
-2
-4
<-5
κ1 (μm⁻¹)
−9.8
0
11.2
>1.6
1
0
-1
-2
-3
<-3.7
Δκ1 (μm⁻¹)
B)
MDSB
JETS
>5
0
-5
-10
-15
-20
-25
<-30
κ2 (μm⁻¹)
−6.6
0
15.6
>5.0
4
3
2
1
0
-1
<-1.8
Δκ2 (μm⁻¹)
C)
MDSB
JETS
>35
30
20
10
0
-10
-20
<-25
κ1 (μm⁻¹)
−251.5
0
66.1
>9.7
5
0
-5
-10
-15
-20
<-25.5
Δκ1 (μm⁻¹)
D)
MDSB
JETS
>15
0
-20
-40
-60
-80
-100
-120
<-130
κ2 (μm⁻¹)
−209.5
0
204.8
>40.0
30
20
10
0
<-8.9
Δκ2 (μm⁻¹)

Fig S1. Comparison of Meyer-Desbrun-Schröder-Barr (MDSB) and Cazal-Pouget (JETS) curvature estimates for the single spine geometry. A) First principal curvature of the plasma membrane. B) Second principal curvature of the plasma membrane. C) First principal curvature of the endoplasmic reticulum. D) Second principal curvature of the endoplasmic reticulum. Shown on the left column are the estimated curvature values. On the right column the difference ($\Delta = \text{MDSB} - \text{JETS}$) between estimates is shown. The difference values are truncated at the 1st and 99th percentiles to improve color range. The distribution and full range of differences are plotted in the inset. Curvature estimates from both algorithms are qualitatively similar. Differences in quantitative value are the greatest at high curvature regions. For the JETS estimate, a jet of second degree was fit to six neighboring points for all meshes.
