# Supplementary material for: 3D mesh processing using GAMer 2 to enable reaction-diffusion simulations in realistic cellular geometries
Source: PLoS Comput Biol. 2020 Apr 6;16(4):e1007756. doi: 10.1371/journal.pcbi.1007756 (PMC7162555; doi:10.1371/journal.pcbi.1007756)
Supplement: S1 Table — (PDF) [file pcbi.1007756.s002.pdf]

3D Mesh processing using GAMer 2 to enable reaction-diffusion simulations in realistic cellular geometries

Christopher T. Lee, Justin G. Laughlin, Nils Angliviel de La Beaumelle, Rommie E. Amaro, J. Andrew McCammon, Ravi Ramamoorthi, Michael Holst, and Padmini Rangamani

Table S2. Local geometry associated with signs of principal curvatures according to our sign convention.

	$\kappa_1 > 0$	$\kappa_1 = 0$	$\kappa_1 < 0$
$\kappa_2 > 0$	Concave ellipsoid	Concave cylinder	Hyperboloid
$\kappa_2 = 0$	Concave cylinder	Plane	Convex cylinder
$\kappa_2 < 0$	Hyperboloid	Convex cylinder	Convex ellipsoid
