# Supplementary material for: 3D mesh processing using GAMer 2 to enable reaction-diffusion simulations in realistic cellular geometries
Source: PLoS Comput Biol. 2020 Apr 6;16(4):e1007756. doi: 10.1371/journal.pcbi.1007756 (PMC7162555; doi:10.1371/journal.pcbi.1007756)
Supplement: S1 Appendix — (PDF) [file pcbi.1007756.s003.pdf]

3D Mesh processing using GAMer 2 to enable reaction-diffusion simulations in realistic cellular geometries

Christopher T. Lee, Justin G. Laughlin, Nils Angliviel de La Beaumelle, Rommie E. Amaro, J. Andrew McCammon, Ravi Ramamoorthi, Michael Holst, and Padmini Rangamani

Appendix S3: Protocols for the generation and comparison of meshes across software

Below we outline the steps used to generate the meshes shown and compared in Fig. 7. The same initial mesh was input into each software: a single dendritic spine which was minimally curated to fix physical and topological issues such as non-manifold vertices and holes in the mesh due to the cutting plane. This mesh is included in Dataset S7. These protocols represent our best faith effort to use each software as intended in accord with the recommended default settings.

TetWild

The latest version of `TetWild` was downloaded from GitHub. The algorithms in `TetWild` can be customized through the adjustment of several parameters; for example ε adjusts the "envelope size" where a smaller value of ε corresponds to better feature preservation. We generated the meshes for Fig. 7C using the default parameters in `TetWild`.

CGAL

`CGAL` version 5.1 was obtained from GitHub. The example tetrahedral meshing code was adapted for our input. The final source script is shown in Listing 1. We note that the criteria for the generated mesh can be tuned by the end-user to modulate the quality of the final product. Here, we have chosen values in accord with the example scripts and the online documentation.

Listing 1. CGAL code used to generate meshes

```
#include <CGAL/Exact_predicates_inexact_constructions_kernel.h>

#include <CGAL/Mesh_triangulation_3.h>
#include <CGAL/Mesh_complex_3_in_triangulation_3.h>
#include <CGAL/Mesh_criteria_3.h>
#include <CGAL/Polyhedron_3.h>
#include <CGAL/boost/graph/helpers.h>
#include <CGAL/Polyhedral_mesh_domain_3.h>
#include <CGAL/make_mesh_3.h>
#include <CGAL/refine_mesh_3.h>

// Domain
typedef CGAL::Exact_predicates_inexact_constructions_kernel K;
typedef CGAL::Polyhedron_3<K> Polyhedron;
typedef CGAL::Polyhedral_mesh_domain_3<Polyhedron, K> Mesh_domain;

typedef CGAL::Sequential_tag Concurrency_tag;

// Triangulation
typedef CGAL::Mesh_triangulation_3<Mesh_domain,
    CGAL::Default,Concurrency_tag>::type Tr;

typedef CGAL::Mesh_complex_3_in_triangulation_3<Tr> C3t3;

// Criteria
typedef CGAL::Mesh_criteria_3<Tr> Mesh_criteria;

// To avoid verbose function and named parameters call
using namespace CGAL::parameters;

int main(int argc, char*argv[])
```

```
{
  if(argc < 3){
    std::cerr << "Required args: domain.off output.off" << std::endl;
    return EXIT_FAILURE;
  }
  const char* fname = argv[1];
  const char* oname = argv[2];
  // Create input polyhedron
  Polyhedron polyhedron;
  std::ifstream input(fname);
  input >> polyhedron;
  if(input.fail()){
    std::cerr << "Error: Cannot read file " <<  fname << std::endl;
    return EXIT_FAILURE;
  }
  input.close();

  if (!CGAL::is_triangle_mesh(polyhedron)){
    std::cerr << "Input geometry is not triangulated." << std::endl;
    return EXIT_FAILURE;
  }

  // Create domain
  Mesh_domain domain(polyhedron);

  Mesh_criteria criteria(
    facet_angle=25, facet_size=0.025,
    facet_distance=0.1, cell_radius_edge_ratio=3,
    cell_size=0.1);

  // Mesh generation
  C3t3 c3t3 = CGAL::make_mesh_3<C3t3>(domain, criteria);

  // Output
  std::ofstream medit_file(oname);
  c3t3.output_to_medit(medit_file);
  medit_file.close();
  return EXIT_SUCCESS;
}
```

Remesh (Hu et al.)

The latest version was downloaded from `https://sites.google.com/view/kaimohu/tvcg_remesh`. To remesh the initial surface mesh we ran the isotropic remeshing workflow with default parameters.

VolRoverN

The quality of the input surface mesh was improved by running 10 iterations of the Level Set Boundary-Interior-Exterior (LBIE) algorithm as implemented in `VolRoverN`. Following this we attempted to tetrahedralize the mesh using the "Tetrahedralize" tool in `VolRoverN`. While an output file was generated, the resultant mesh only had 362 tetrahedra and 305 nodes, with most of them being disjoint. The version used was the Mac binary, `VolRoverN-2.0.6909.dmg`, available at `https://cvcweb.oden.utexas.edu/cvcwp/software/volumerover-neuron` at the time of writing.

GAMer 2

The mesh conditioning operations were applied to the initial meshes using `BlendGAMer`. Once the surface mesh had suitable quality, it was passed to `TetGen` for tetrahedralization. We note that `TetGen` is asked not to modify the surface meshes when tetrahedralizing.
