# Supplementary material for: 3D mesh processing using GAMer 2 to enable reaction-diffusion simulations in realistic cellular geometries
Source: PLoS Comput Biol. 2020 Apr 6;16(4):e1007756. doi: 10.1371/journal.pcbi.1007756 (PMC7162555; doi:10.1371/journal.pcbi.1007756)
Supplement: Attachment — Submitted filename: response.pdf [file pcbi.1007756.s007.pdf]

Response to Reviewers:

We thank the reviewers for their extensive, diligent, and insightful comments. We have responded to specific concerns below and modified the manuscript accordingly; changes in the manuscript are highlighted in red in a separate PDF file attached for review only. The reviewer's comments are given in **bold face** with our response in plain text. Where appropriate, portions of the manuscript that are changed are provided.

Reviewer 1:

The paper from Lee et al. explores specifically one problem, the generation of meshes from segmented EM micrographs, which is usually good enough for visualization using standard algorithms, but needs to have certain constraints if scientists want to use them for simulations or morphological analysis. Authors state to have developed a full pipeline bringing from 3DEM to volume meshes including GAMer2, a mesh processing software. Although the problem tackled is of high importance in the field, some points came directly to my attention during the reading of the manuscript.

i) Figure 1 shows the main steps of their pipeline. But reading the manuscript further, it is not entirely clear to me whether they first generate the meshes from segmentation software, and then use GAMer2 to improve them, or they rather use the segmentation itself to generate the mesh. I think this is important to clarify. It indeed rather seems that authors are first generating the meshes and then fixing it, staying to the Mesh Generation Pipeline (line 226). Authors seems to have used IMOD in their test case, which generates mesh starting from contours of segmented micrographs, while most users from the 3DEM community rather generate mesh from the masks extracted from tools such as TrakEM2.

The reviewer is correct in their assessment that the meshes are generated from segmentation software and then input into `GAMer 2` for improvement. To clarify this point, we have modified Fig. 1 to highlight the steps handled by `GAMer 2` in the overall workflow; specifically, the mesh conditioning in going from the input in Fig. 1C to the optimized mesh in Fig. 1D. We have also rewritten portions of the manuscript to highlight the input and output of all steps.

In this test case, the dataset and segmentation were performed using IMOD by Wu et al. We note that `GAMer 2` is a general mesh generation and conditioning tool capable of reading in several popular unstructured mesh file formats[1]. Moreover the `BlendGAMer` add-on benefits from `Blender`'s capabilities to read many other file formats. We are confident that meshes generated using the TrakEM2 workflow[1] are compatible with `GAMer 2`.

ii) The authors state at the very beginning out loud to propose a whole pipeline, from image segmentation to simulation. Actually, the whole point of the paper, is about the remeshing, and how producing healthy meshes is critical for simulations and analysis. This is really good, but authors might want to reconsider statements in the abstract and introduction where they say that this paper provides a whole pipeline for image processing. They do produce meshes in several ways, from different available image segmentation software, that they do not describe, therefore these should not be considered part of the method.

In accord with both this comment and those of Reviewer 2, we have modified the claims made throughout the paper to highlight the remeshing capabilities of `GAMer 2`. We have also moved sections discussing the meshing pipeline from the methods to the results as suggested by Reviewer 2 to distinguish our main contribution.

iii) Lines 1 to 18 are completely devoid of literature citations. Authors could cite the review from Knott and Genoud "Is EM dead?", the pioneering work from Denk and Horstmann, PLoS Biology, 2004 showing the first 3View setup, on top of my head. Also, they should cite and make some examples of few works showing how 3DEM and image segmentation have helped

[1] `https://imagej.net/TrakEM2_Scripting#Generate_3D_meshes`

to visualize the three-dimensional structure of entire cells, and their content. For instance the Cell paper from Kasthuri et al., 2015, or the PLoS one paper from Calì et al., 2018, where authors shows dense reconstructions of neuropil, including mitochondria and synaptic vesicles. I think all these examples helps to contextualize their work, and authors should make a careful literature review and cite properly the sources.

We thank the reviewer for bringing this to our attention. We have added additional citations to relevant Volume EM literature as well as to several example datasets to help contextualize our work.

iv) Blender sculpt mode is mentioned. This is just a curiosity, but does authors use sculpt to fix the meshes? It is dangerous as a smoothing tool, given that arbitrary modifications of the surface might lead to change in SA/Vol ratios, and most likely change the Surface Area of the original mesh. Authors should discuss carefully this point. This also leads to the question, whether authors are also considering to test their meshes for preserving their volume and more importantly surface area, which is sensitive to mesh manipulations.

We acknowledge and agree with the reviewer's point that manual sculpting can be a dangerous tool for smoothing. In our current approach the sculpting tools are only used to correct major topological errors and very bad non-manifold features. For example, in the segmentations of the ER, tubules are often disconnected while the micrograph suggests otherwise, see modified Fig. 2. We have chosen to perform manual curation to reconnect the tubule. Where appropriate we have modified the main text to distinguish between manual modifications and automated `GAMer 2` operation.

We have added quantification of the volume and surface area before and after processing to Table 1. We find that there is reasonable quantitative agreement of the volumes before and after processing. On the other hand, the surface area of the meshes is reduced in all examples. This is, in part, due to the smoothing of jagged boundaries along the z-axis from misalignments in the segmentation.

To help motivate the need for and to distinguish between manual curation and `GAMer 2` operation, we have added a section reporting topological Betti numbers, Table 2. In this work, we have performed manual curation to connect disconnected membranes which we believe should be continuous. Also manual curation was applied to resolve some non-manifold artifacts such as edges connected to three or more faces. We note that some advanced meshing codes such as `TetWild` [2] are robust to non-manifold artifacts. Others such as Hu et al. Remesh[3], `VolRoverN` [4], and earlier versions of `GAMer` may crash without warning. `GAMer 2` and `CGAL` 3D Mesh Generation[5] will throw an error.

v) going through the methods, which are convincing and very well documented, I think one very important thing to include is whether you can proofread the morphology of the mesh by superimposing the mesh itself with the original image stack. Although qualitative, it is an important thing to check, and would be good to incorporate in a figure.

We thank the reviewer for this suggestion. One can indeed proofread the morphology of the mesh by superimposing the mesh with the original image stack. We have included two supplemental movies (Movies S2 and S3) proofing the initial and final meshes against the original image stack for the one spine geometry.

vi) line 239, authors state that quantities like surface areas, volumes and curvatures can be estimated, and then correctly document how to estimate curvature, which is of interest for biophysicists. Nevertheless, surface area and volume are quantities of interest as well, especially for biologists. In both cases, I wonder whether authors accounts at all for renormalization in the order of magnitude of the image stack (nanometers or micrometers), or the simply keep measurements as arbitrary units. This is really important, as simulations then use constant and variable that are bind to proper physical units.

We thank the reviewer for this question. Indeed we do scale the initial meshes to correspond to some physical unit. The following text has been added to describe the scaling performed in our applications:

"The output initial meshes have units of pixels and were scaled to µm using an 8 nm isotropic voxel size."

Although not present in our examples, some imaging modalities have anisotropic resolution or samples can suffer from directional shrinkage. It is also possible to correct for this by scaling the mesh by different values

along specific coordinate directions.

Finally, I am fully convinced of the added value of the meshing tool that the authors provide, and how the meshes are proper and suitable for physical simulations; nevertheless, I am far less convinced (because I don't think this is properly documented) of the degree of fidelity of the meshes compared to the real morphology, also given that authors seems to fix artefacts manually using blender tools, without proofreading the meshes together with the original image stack. I think authors should carefully address this point in particular.

We thank the reviewer for their positive assessment of our work. To address the similarity between the final meshes and the input micrographs we have added Supplemental Movies S2 and S3. While the end-user can use Blender tools such as sculpt mode in a diabolical manner, when carefully applied, the final result will be similar to the initial input, as demonstrated. We note here that some changes, such as the manual connection of two disjoint ER segments, will fundamentally change the overall topology. While this causes deviation from the "ground truth", defined as a segmented micrograph, connectivity of compartments is important and in many contexts, other experiments may suggest to a trained biologist that the disconnection is an artifact from either incorrect segmentation or even insufficient resolution by electron microscopy. We anticipate that as imaging modalities and machine learning based segmentation algorithms improve, the need for manual curation will decrease.

Reviewer 2:

This manuscript introduces GAMer 2, a mesh processing tool which takes surface meshes reconstructed from EM images as input, and produces tetrahedral meshes that are suitable for computational simulations. This manuscript further describes a pipeline solution from EM imaging of cellular structures to simulations with meshes of these structures, by utilizing GAMer 2 as well as other third-party tools such as IMOD, Blender, TetGen, and FEniCS.

Unfortunately, the title, abstract, and some texts in this manuscript are inaccurate and misleading. GAMer 2, as at the current stage, does not directly operate on cellular electron micrographs as described in the title, but on the surface meshes generated from third-party tools like IMOD. This manuscript does not demonstrate sufficient contribution to the image acquisition, segmentation, and the initial surface mesh generation steps of the described pipeline (A to C of Fig 1). The third-party software packages used in the pipeline steps are some of the common choices for the procedures, and the usages described in the manuscript seem to be generic. Furthermore, similar workflow from contour tracing data to simulation-ready tetrahedral mesh has been introduced in at least one of the referenced publications ([10] Edwards and etc., 2014) where all steps of the pipeline were integrated into a single application. With comparison, which should have been done in the manuscript, the claim of "develop an end-to-end pipeline for this task" seems to be overstretched and should be rephrased. It is acceptable that a manuscript presents the functionalities of the software and demonstrates its application by presenting a pipeline scheme as an example. However, the contribution of the presented software and any novelty in the pipeline steps should be stated without ambiguity. The author should avoid emphasizing general usage of a third-party software package in a pipeline step as novel development, particularly when the step is not performed by the author, or the third-party software has not been integrated into the author's software.

We thank the reviewer for this comment. We have made major modifications to the text to emphasize our main contribution, `GAMer 2`, while removing the claims of developing an end-to-end pipeline. In the revision process, we have taken the reviewers' advice to present the functionalities of `GAMer 2` followed by demonstration and application. As noted in our earlier response to Reviewer 1, Fig. 1 now makes clear the mesh processing step actually performed by `GAMer 2`.

Despite the inaccuracy of the description, the software presented in this manuscript does contribute to the computational biology community by bridging the gap between surface mesh

reconstruction of cellular structures via EM imaging, and simulation-ready mesh generation. Surface meshes generated from EM image tracing and segmentation applications are commonly for visualization only, thus inadequate for the use of computational simulation due to problems such as manifoldness. Progress has been made in recent years as referenced in the manuscript, but there remains no widely accepted, efficient solution for the problem. The software presented here can be considered as a novel candidate. The pipeline example in the manuscript also demonstrates the application of this software for common modeling interests in the field, providing workflow guidance for future research.

We thank the reviewer for recognizing and acknowledging the contribution of our work.

Detailed Comments

Title:

See General Comment.

The title has been changed to: "3D Mesh processing using GAMer 2 to enable reaction-diffusion simulations in realistic cellular geometries"

Abstract:

"we develop an end-to-end pipeline for this task" see general comment.

The abstract has been updated to focus on `GAMer 2` as suggested by the reviewer.

"We apply this pipeline to a series of electron micrographs of neuronal dendrite morphology" According to Line 227, the starting point of the pipeline should be the contour reconstruction of the segmented micrographs rather than the micrographs themselves.

We have updated the abstract to reflect the actual start and endpoints of `GAMer 2`.

"In this paper, we describe the use of our recently rewritten mesh processing software, `GAMer 2`, to bridge the gap between poorly conditioned meshes from segmented micrographs and simulation ready models."

Author Summary

"Here, we describe a pipeline that takes images from electron microscopy as input ..." See comment above.

The author summary has been updated to emphasize the role of `GAMer 2` in the example applications.

"Using a workflow consisting of other open-source softwares along with `GAMer 2`, we demonstrate the process of going from electron micrographs to simulations for several scenes of increasing length scales."

Introduction

The introduction section needs to be reorganized. At the moment, its focus switches back and forth between GAMer2 and the pipeline, mixing actual contribution from the author with third-party supports. A reasonable structure would be first to present the pipeline as a potential solution from EM imaging to computational simulation, then provide a general review on the state-of-the-art tools and solutions for each step, highlight the challenges faced in practice, finish with stating the novel contribution of this work to this pipeline, either in software development or modeling.

We thank the reviewer for this helpful suggestion. The introduction has been rewritten to follow the structure as suggested. In our concluding paragraph, we highlight our motivation and objectives for developing `GAMer`

2.

Line 72: "as shown in Fig. 2." It is better to move Figure 2 to the "Meshing segments of increasing length scales" section so that the readers do not need to switch between Figure 2 and Figure 5 constantly. As here is a general introduction of mesh artifacts, referencing Figure 2 is not necessary — the same for line 76.

Fig. 2 has been remade and references in the text around Fig. 5 no longer reference Fig. 2.

Methods:

The author should clarify how GAMer2 differs from its previously published predecessor in terms of mesh processing algorithms, as now there seems to be a mix between GAMer and GAMer2 functionalities. If there are new contributions or significantly different from previous designs, the author should provide a clear statement here. Also, if the author decides to use GAMer as the simplification of GAMer2 in the rest of the manuscript, there should be a clear statement.

We have added an explicit subsection entitled "GAMer 2 Development" which differentiates between `GAMer` and `GAMer 2`. Throughout the text, we now say `GAMer 2` to refer to the latest version and `GAMer` otherwise.

Line 109: "Finite elements simulations" → "Finite element simulations" "the quality of the mesh" → "mesh quality"

Done.

Line 111: "triangulation with high aspect ratios..." → "triangles with high aspect ratios..."

Done.

Line 114: "This scheme is an extension ..." This sentence is difficult to read. The author should try to rephrase it.

The sentence has been rewritten to: "This scheme is an extension of the angle weighted smoothing scheme, formulated for 2D and described by Zhou and Shimada, to three dimensions."

Line 138: "These images can produce meshes that ... making it particularly suitable for cellular images." This is not very convictive that the quantity of mesh elements is the cause of the failure of global optimization. As commented above, this work does not directly operate on the cellular images but their post-processed products, whose quality relies on the solutions of previous steps. One may argue that the suitability of the smoothing algorithms is simply the consequence of tool selections at previous steps, rather than the nature of the images.

We thank the reviewer for making this point. In this revision we have, throughout the text, added additional discussion on how `GAMer 2` differs from other existing mesh generation codes. We have also added Fig. 7 which compares the `GAMer 2` result against other codes. In the section presenting the Fig. 7 result, we discuss the preservation or smoothing of fine features. We suggest that many of the fine features observed may be artifacts of image alignment and registration. However, we note that we make no claims on the validity of either approach (smoothing or preserving fine features). The validity of each approach should be compared in the context of a real experimental validation, which we feel is beyond the scope of this work.

Line 156: What is the reason for putting the decimation section between two smoothing algorithms?

We thank the reviewer for their suggestion and have swapped the order of appearance for decimation and normal smooth sections such that the two smoothing subsections appear back-to-back.

Line 165: "finite elements simulations" → "finite element simulations"

Done.

Line 168: "topology changing" → "topology-changing"

Done.

Line 221: The author should provide a figure to demonstrate the usage of BlenderGAMer for boundary marking.

We have added Fig. 5 to the methods showing the usage of `BlendGAMer` for boundary marking.

Line 226: "Mesh Generation Pipeline" This paragraph relates to a specific example instead of the general method, thus should be put in Results instead.

We have moved this paragraph to the Results section.

Line 254: The author needs to clarify if the modeling work presented in the manuscript provides a novel solution to the problem, or is a reproduction of results for the validation of conditioned meshes processed by GAMer2. If it is the first case, then the work presented here establishes a pipeline from mesh processing and boundary marking to modeling with techniques specific to the conditioned meshes. If it is the second case, however, this work should not be regarded as pipeline development, but an application demonstration of the processed meshes. And it should be put to the Result.

The modeling work presented in the manuscript serves as a validation of the conditioned meshes generated using `GAMer 2`. Text describing this work has been moved to the Results section accordingly.

Line 323: "NMDAR" and "NMDA receptor" are both used in the manuscript, and should be unified.

Done.

Line 352: "We demonstrate the application of our pipeline to dendritic spine reconstructions of different sizes." See general comment.

We have removed the mention of pipeline development.

Line 396: "the distribution of the angles of the surface mesh" → "the distribution of the triangular angles of the surface mesh"

Done.

Line 405: "but is easily and automatically resolved" → "but is easy to be resolved automatically"

Done.

Line 398: "Prior to conditioning, the angle distribution is spread out and contains many large and small angles" A quantitative result is preferred. The author can present the means and standard deviations of both distributions for better comparison.

We have added quantification of the standard deviations before and after processing with `GAMer 2`. Since we are computing the distribution of interior angles of triangles, the mean will always be 60 degrees ($180 * N/(3 * N) = 60$, where N is the number of triangles). Therefore we do not report the mean value.

Line 407: "the angles of the mesh" → "the angles of triangles of the mesh"

Done.

Line 409: "it may be necessary to increase the number of triangles to accurately capture the fine details." All geometry details exist in the initial mesh reconstruction, so this statement is not accurate. → "it may be necessary to increase the number of triangles to accurately capture the fine details with high mesh quality."

Done.

Line 434: "and the PSD is marked with GAMer for use denoting a boundary condition." GAMer or BlenderGAMer?

The boundary marking is performed using `BlendGAMer` in this case, although `libGAMer` can also be scripted to perform boundary marking. The text has been updated to reflect this.

"Boundaries were marked using `BlendGAMer`, although `PyGAMer` and `GAMer 2` can be scripted to assigne boundary labels as well, and the conditioned surface meshes were tetrahedralized using `TetGen`"

Line 444: "using methods from discrete differential geometry." The author can refer to previous Method section.

Since this is an application, the methods have been consolidated with the main Result text.

Line 444: "Shown in Figs. 6 and 7 are the principal curvatures $\kappa 1$ and $\kappa 2$ respectively" → "The principal curvatures $\kappa 1$ and $\kappa 2$ are shown in Fig 6 and 7, respectively."

Done.

Line 449: "We also observe that tubular regions such as the neck of the spine have near uniform curvature." The curvature values of the neck region are truncated in the colormap, so this conclusion can not be made from the current result, the same for the ER neck result. The author should analyze the values of the spines in the dendrite segment example to form a population result.

We agree with the reviewer that a population result would be superior. However, owing to the points brought up by Reviewer 4, specifically on the uncertainty of curvature estimates from polygonal meshes, we feel additional care should be taken before we make any conclusions regarding the values of curvature. We have thus removed the offending claims about the uniformity of curvature values along the membranes. In future work, we will work on the robustness of curvature estimation and produce an ensemble result.

The author can also calculate the principal curvatures of the initial meshes or the conditioned meshes with different smoothing and decimation thresholds, and compare their results with the current ones to identify if these characteristics are consistent features of the cellular structures or the artifacts from the meshing process.

We agree with the reviewer that this would be an interesting and valuable experiment in principle. However, as highlighted by Reviewer 4, estimators of curvature on discrete meshes are often sensitive to the mesh quality and even properties such as the vertex adjacency. To perform a careful analysis of the change in the estimated curvature throughout the remeshing process will necessitate the use of other robust algorithms or approaches capable of producing accurate curvature estimates on poorly conditioned meshes. Owing to the uncertainty in the estimated curvature values, we have omitted any strong claims about the curvature in the manuscript.

Line 464: "Simulations of Surface Diffusion" This is different from the "Coupled volume and surface diffusion model" simulation presented before. Can the author clarify it, please?

The Methods and Results sections have been streamlined as suggested by the Reviewer which we believe resolves this issue.

Nevertheless, the text has been clarified to differentiate between the different simulations we ran. We describe two separate models: 1) $A + X \longleftrightarrow B$ and 2) calcium ion signaling. In the first model, the surface finite element method is used to model a reaction between a volume component A and membrane bound component X to produce membrane bound B. In the second model, calcium influx through NMDAR is modeled as a boundary flux. This distinction should be clear in the new organization.

Line 466: "we simulated the reaction of a volume component A reacting with membrane bound species X to form membrane bound species B." This should refer to previous sections of the model rather than repeating again.

The Methods and Results sections have been streamlined as suggested by the Reviewer which we believe resolves this issue.

Line 468: Why Figure 8 appears after Figure 9 in the main text?

The manuscript text has been reorganized and the figure order has been double checked.

Line 485: "GAMer 2" the name and version of the software should be consistent. This section should be put after the model examples. The author may also explore the scenario where the

surface mesh remains the same while the tetrahedral mesh is progressively refined by TetGen, and compare the result.

In the reorganization, we have placed the "Mesh Convergence Analysis" after the "Simulations of coupled surface and volume diffusion model" section but before the "Simulation of reaction-diffusion equations on a dendrite" section. This is since the mesh convergence analysis is performed on the single spine geometry using the coupled surface-volume model and not the calcium signaling model. Text has been added to emphasize this distinction.

Line 508: A reference to the previous model section is required. Alternatively, the previous section can be relocated here.

The previous section has been relocated to the Results.

Line 543: "...that result from our tools are high quality" → "...that result from our tools are of high quality"

Done.

Line 544: "in finite elements simulations" → "in finite element simulations"

Done.

Line 574 Line 579: This sentence requires to be rewritten.

This sentence has been updated to refer to our contribution, `GAMer 2`.

"Specifically, we anticipate that models enabled using `GAMer 2` will be of significant interest to two broad communities in computational biology – membrane biophysicists focused on the analysis and simulation of membrane shapes, curvature generation, and membrane-protein interactions and by systems biologists focused on understanding how cell shape and internal organization can impact signal transduction and the dynamics of second messenger microdomains."

Conclusion:

GAMer 2 should be mentioned in the conclusion, as it is the main contribution.

The conclusion has been modified to mention `GAMer 2`.

Figures:

Fig. 1: This figure needs a complete rework as commented in the General Comment.

We have updated this figure and the associated caption to reflect the main contribution of this work, `GAMer 2`. We believe that the figure retains value as an introduction to the workflow steps necessary to go from images to simulations. To avoid misrepresentation of the work, we have placed a box around the steps which are handled by `GAMer 2` in the figure.

Fig. 2: As mentioned above, it may be better to relocation this figure. "Left, due to the fine ultrastructure of the process, portions of the ER are not resolved by the microscope and become erroneously disconnected as separate meshes." Alternatively, this could be caused by human error during the contour tracing process and/or insufficient triangulation solution used for surface mesh generation. The conclusion can not be made without presenting the original images as well as the contour tracing data.

Fig. 2 has been updated to show a proof of the initial single spine mesh against the original micrographs. We speculate that the disconnections of the ER are often due to both insufficient resolution of EM and errors in human driven segmentation. Fig. 2 C and D provide an impression of the difficulty of the task and justify our speculation.

Fig. 4: "weighted and unweighted meshes" according to the figure legend as well as the order of previous subfigures, "weighted" and "unweighted" should be switched.

We thank the reviewer for highlighting this point of confusion. We have updated the color scheme in the histogram shown in panel C to match with the meshes in panels A and B. The caption of this figure has also been updated to more clearly reflect the differences.

Fig. 5: As mentioned above, more quantitative results are required in the explanation text such as the means and standard deviations.

As aforementioned we have computed the standard deviations of the distribution of angles. This information is reported in the figure caption. The mean of these distributions will always be equal to 60 and is therefore not reported.

Fig. 6: "B) two spine moddel, left" → "B) two spine model, left"

Done.

Fig. 11: A summary of how the morphology affects the calcium dynamics is needed.

We have added additional discussion to the figure caption summarizing trends in how the morphology affects the calcium ion dynamics.

The inconsistency of font sizes and figure styles (in particular, Fig, 4C, 8F and 11) makes the read of figures slightly uncomfortable.

We apologize for this. All figures in this work have been remade to match font sizes as appropriate.

Reviewer 3:

The manuscript describes a system of algorithms and software implementations of 3D mesh processing and then applies it to generating high-quality meshes from cellular electron micrographs. Although the algorithms are mostly taken from previous work, the present work is timely and valuable for its novel implementation of the methods and public availability of source code. The manuscript is very nicely written and the results are properly shown and discussed. I would suggest a minor change in the result section:

The authors show how mesh quality is improved after mesh processing (specifically, mesh smoothing). However, the ultimate goal of mesh processing in the current work is to provide high-quality tetrahedral meshes used for finite element analysis. For that reason, I would suggest the authors conduct some quality analysis of the tetrahedral meshes as well. For instance, one may see the tetrahedral meshes get improved while the surface meshes are being smoothed.

We thank the reviewer for their positive assessment of our work. We have incorporated this suggestion to quantify the quality of the tetrahedral mesh in response to `GAMer 2` mesh conditioning into Fig. 11. Indeed, as predicted by the reviewer, the quality of the tetrahedral meshes do indeed improve as the surface meshes are conditioned.

Reviewer 4:

Starting from electron microscopy micrographs, this paper presents a pipeline reconstructing 3D meshes amenable to Finite Elements simulations. Developing such a pipeline is an important and timely endeavor, as the tool provided should foster our understanding of the structure to function relationship, at length scales ranging from micro to nanometers.

While the paper is of interest, I was not convinced for three reasons: 1) the specific difficulties dealt with are not carefully specified, 2) the individual steps undertaken do not use state of the art methods, 3) the assessment is not careful enough in terms of base-line. The software development undertaken is very significant, and it could be that the pipeline proposed is if very significant interest for biologists. Yet, this has to be documented more carefully.

Major Points:

As detailed below, I see two major problems with this paper.

Ad hoc methods:

Starting from raw slices of electron microscopy data, the proposed method involves the following steps: contour segmentation, primitive mesh reconstruction from stacked contours, meshing and smoothing.

The problem is that for each of this step: (i) the problem tackled is not formally defined – the type of guarantee sought is not documented, (ii) previous work is not adequately discussed, and (iii), in several cases, the methods used are not state of the art.

Thus, overall, the proposed method is more of an ad hoc pipeline, rather than a sequence of carefully optimized steps.

We thank the reviewer for raising these important points. In this revision we have added text defining the challenges faced by the biophysics community seeking to produce models from electron micrographs. While we agree with the reviewer that using state-of-the-art methods with provable guarantees would be ideal, we posit that the some guarantees such as strict input geometry preservation will produce results that are not representative of the underlying biology. For example, in biological contexts, compartmental connectivity is important. Disconnects in an organelle such as the ER can lead to incorrect estimates of values such as transport rates through the ER network. Owing to the poor signal-to-noise ratio of EM datasets, as illustrated in Fig. 2C and D, without additional biological context, it is often difficult to define an objective ground truth. The unfortunate state-of-the-art in biophysics remains dependent upon intervention by trained biologists. To this end, we sought to develop a general and user friendly software to facilitate usage by life sciences researchers.

With respect to mesh generation performance, Fig. 7 illustrates that our process results in meshes with quality comparable to several state-of-the-art alternatives. For this comparison, manual curation was used to produce a watertight and manifold surface mesh which was use as the input. No codes except `TetWild` were capable of operating on the initial mesh produced by `imod2obj`.

We recognize that the sequence of methods described in our approach may not be ideal. Furthermore we acknowledge that the choice of methods for early workflow steps can contribute to difficulties at later workflow stages. Nevertheless, the rationale for our choice of methods is that, in many cases, we are restricted to softwares which are both publicly available and interoperable with the outputs of other workflow steps. We believe that this lack of code availability and data interoperability is a major limitation to the field. To this end, in future work, we aim to use `GAMer 2` as a platform to incorporate advanced algorithms from other disciplines. As an example, `TetWild` is capable of producing amazing results in an automated manner. However, to the best of our knowledge, `TetWild` does not support the definition of region and boundary marked values which are necessary for the definition of initial and boundary conditions in the subsequent simulation steps. Furthermore, `TetWild` seeks to preserve the geometry of the input triangle soup to within some user specified deviation, ϵ, which is usually set to a small value. While feature-preservation is desired, some of the features of the input mesh may be artifacts of earlier workflow steps. In this work, we have chosen to perform surface mesh fairing prior to tetrahedralization to remove some of the artifacts resulting from poor image alignment. We view `GAMer 2` as a potential platform for the integration of these packages and we fully intend to seek out collaborations with experts in mesh generation from other communities in our future work.

Significance of the methods proposed for the problems at hand:

In several places, the authors discuss the intrinsic difficulties of the problems tackled, for instance:

“However, in subcellular scenes where the geometry may be tortuous and local receptor clusters can be arbitrarily distributed on the manifold, boundary definition is a non-trivial challenge.”

“As noted earlier, finite elements simulations are sensitive to angles of the mesh. ”

“In addition to the generation of FEA compatible meshes of realistic cell geometries...”

Unfortunately, this specification is rather vague. I would urge the authors to focus on selected challenges such as “volume conservation of organelles”, “preservation of thin parts in the reconstruction / meshing”, “enforcement of boundary surfaces via constrained/conforming Delaunay triangulations”, “enforcement of bounded principal curvatures on boundary surfaces”, etc, so as to stress the added value of the proposed pipeline.

Without a clear identification of such challenges, making strong claims is hardly possible.

We have added additional text to the Introduction discussing our main design criteria in developing `GAMer 2`. Owing to the unfortunate need for human curation of datasets, we have developed a user friendly code. Our main contribution is the production of a code capable of taking surface meshes and producing a boundary marked and annotated surface or tetrahedral mesh which is compatible with finite element codes such as `FEniCS`, that is usable by the general biophysicist.

Details:

page 4. About “However, to the best of our knowledge, there is no current free and open-source system for going from EM images to high-quality 3D meshes, which are essential for developing reliable high-resolution finite element simulations of cellular processes.” If one takes for granted the contours computed from the segmented micrographs, there exists very high quality 3D meshing packages, in particular the one from CGAL: see `https://doc.cgal.org/latest/Mesh_3/index.html`

We agree with the reviewer that there exist many high quality 3D meshing packages. We have added a comparison of meshes produced by several of these codes in Fig. 7. We found that for some codes such as `VolRoverN`, Hu et al. Remesh, and `CGAL` Mesh 3, many input meshes would produce segmentation faults. After manually curating these initial meshes, much like was performed in our workflow, we were met with mixed results. To the best of our knowledge, none of the other codes tested support the association of metadata such as boundary markers in their outputs. `VolRoverN` failed to produce a tetrahedral mesh. For `CGAL`, we attempted to produce a tetrahedral mesh of the ER within the PM by adapting the included examples to read in our PM and ER meshes but were unsuccessful.

In future work, we plan to connect `GAMer 2` to other tools such as these, bringing accessibility to the broader mesh generation tool corpus.

*** page 4. Surface reconstruction from cross sections. Many provably correct reconstruction methods from cross-sections have been developed, starting with the seminal work exposed in Boissonnat, Jean-Daniel, and Bernhard Geiger. “Three-dimensional reconstruction of complex shapes based on the Delaunay triangulation”. Biomedical Image Processing and Biomedical Visualization. Vol. 1905. International Society for Optics and Photonics, 1993. The following paper presents a mini review of recent work on this topic: Bermano, Amit, Amir Vaxman, and Craig Gotsman. “Online reconstruction of 3D objects from arbitrary cross-sections.” ACM Transactions on Graphics (TOG) 30.5 (2011): 113.**

We thank the reviewer for pointing out this literature on surface reconstructions from cross-sections. Unfortunately when dealing with noisy micrographs, the segmentations and labels can rarely be taken as is; errors due to the imaging modality can further contribute to uncertainty in the data. We posit that for biological specimens such as the endoplasmic reticulum, where even the highest resolution imaging modalities may fail to capture its geometric intricacies, constructing the best approximation of a biophysically realistic mesh will often require human intervention. Thus, our efforts have primarily been focused on developing a

user-friendly tool to encourage biology experts to contribute their domain knowledge towards the generation of realistic meshes. With that being said, we certainly agree with the reviewer that the use of provably correct algorithms is both appropriate and ideal. Since the algorithms described in `GAMer 2` are general for any surface mesh, meshes produced by other approaches such as that described by Bermano et al. would be compatible with `GAMer 2`. Moreover, for the methods described by Bermano et al. and Boissonnat and Geiger, a software or library implementation could not be found through Google searching and visiting the authors webpages.

page 5. About "In order to enable modeling using the shapes represented by the contours, geometric meshes compatible with numerical methods can be constructed". please clarify the methods you have in mind, as specific methods typically impose different requirements on the meshes.

We have included additional discussion in the introduction about the specific methods we have in mind.

"Popular biophysical simulation modalities such as finite element method[6, 7], particle-based stochastic dynamics[8, 9, 10, 11], and reaction-diffusion master equation[12, 13, 14, 15, 16, 17, 18] among many others require discretizations or meshes representing the geometry of the domain of interest. Moreover, in biological systems, the localization of molecular species is often heterogeneous which necessitates the need for boundary and region marking to represent this heterogeneity."

page 6. About "We note that although there exist advanced tetrahedral mesh generation tools, such as TetWild [11], which can generate Finite Element Analysis (FEA) compatible volume meshes automatically from these poor quality initial surfaces, many mesh defects are the result of the limited resolving powers of EM (e.g., Fig. 2A1, A2) and require more careful curation". This is a typically example where you should be more precise to specify the defects: are you talking about topological defects (presence of gaps - cracks, voids), or geometric defects (typically poorly shaped simplices such as triangles with obtuse angles or tets such as slivers)?

We have elaborated upon this sentence and updated Figure 2 to illustrate an example of a mesh defect arising from error in the segmentation process. Figure 2C and D show two disconnected segments of ER, although there is no ground truth, the raw EM data shows a shadow over where the contours ought to be if the ER was in fact connected. Based on biological intuition and comparison with the raw data, a biologist can easily curate defects such as these using the `GAMer 2` platform.

"We note that although there exist advanced tetrahedral mesh generation tools, such as TetWild, and others, which can generate FEA compatible volume meshes from these poor quality initial surfaces, these tools are general purpose and currently not adapted to the length scales of single cells and subcellular structures. Mesh defects such as disconnects in the ER arising from the limited resolving powers of EM or errors in segmentation (e.g., Fig 2C, D) require more careful and often manual curation."

page 6. About your meshes. you should describe more carefully the type of mesh you wish to build in order to accommodate the boundary surfaces stemming from the segmentation steps. in particular high quality meshes are typically related to the Delaunay triangulation, which is not mentioned once in your main text.

We thank the reviewer for bringing up this important point. Our main contribution to this work is not in algorithm development but rather creating an easy to use mesh generation platform for biophysical applications. We refer the reader to the earlier `GAMer` literature[19] which describes the algorithms along with any quality guarantees. It should be noted that `GAMer 2` passes its refined surface meshes to `TetGen`, which uses constrained Delaunay triangulation, in order to construct the final tetrahedral mesh. We emphasize that our goal is to provide a tool which allows the common biophysicist to generate a mesh which we believe is both biologically and physically accurate and compatible with finite element simulations.

page 7. About the estimation of normal vectors ie "Vertex normals are defined as the weighted average of incident face normals." It is well known that the estimation of normals and principal curvatures should be undertaken in one step – and not two as yo do, as proved in the following paper Cazals, Frédéric, and Marc Pouget. "Estimating differential quantities using polynomial fitting of osculating jets." Computer Aided Geometric Design 22.2 (2005): 121-146. The

corresponding software is available in CGAL, see `https://doc.cgal.org/latest/Jet_fitting_3/index.html`

We thank the reviewer for bringing this work to our attention. The use of weighted average face normals is common in graphics applications due to the simplicity of this calculation. Since the Local Structure Tensor only requires the normals of each vertex and not the principal directions, the weighted average of face normals is faster to compute. The resulting surfaces produced by `GAMer 2` also reasonably preserve the underlying geometry as illustrated by Supplementary Movie S3 which proofs the final mesh with the micrographs. Nevertheless, we have implemented the algorithm of Cazals and Pouget into `GAMer 2` and the computed normals can be used at the discretion of the end-user.

page 8. About mesh smoothing. For recent advances in mesh smoothing, see the following paper, which addresses 3 challenges at once namely (i) geometry preservation (ii) coarsening and (iii) removal of badly shaped elements: Hu, Kaimo, Dong-Ming Yan, David Bommes, Pierre Alliez, and Bedrich Benes. "Error-bounded and feature preserving surface remeshing with minimal angle improvement." IEEE transactions on visualization and computer graphics 23.12 (2016): 2560-2573.

We thank the reviewer for bringing this work to our attention. We have added a comparison of various algorithms, including the one described in Hu et al. in Fig. 7. We note the excellent performance of the algorithm from Hu et al. in terms of improving surface triangle angles. In fact, the mesh it produced had the greatest minimum angle amongst all other algorithms tested. Despite this performance, the implementation obtained from the author's website has some major limitations. In addition to only providing a Windows binary, we found that the code was not robust to problems with the input mesh "cleanliness". For many of our initial meshes, the software would crash without warning. After manually cleaning the meshes using an external tool (`Blender`), the approach by Hu et al. produced a mesh with statistics reported in Fig. 7. Owing to their use of a strict bound on approximation error, high-frequency artifacts from misalignment of the segmentations are preserved. Therefore, while the algorithms described by Hu et al. are noteworthy, we feel that they cannot be used out of the box and must be adapted for use with `GAMer 2` in future work.

page 12. About boundaries i.e. "However, in subcellular scenes where the geometry may be tortuous and local receptor clusters can be arbitrarily distributed on the manifold, boundary definition is a non-trivial challenge". The classical methods to impose pre-defined boundaries consist of using constrained and/or conforming Delaunay triangulations, see again the aforementioned CGAL packages. please comment the output of your method wrt these classical ones.

We appreciate the reviewers raising this possible point of confusion. As shown in Fig. 5, boundary marking in our work refers to the labeling of subsets of faces on a surface to be later used for defining boundary conditions. For example, in the calcium signaling simulations, the post synaptic density (PSD) where NMDA receptors are localized are the only regions where J_{NMDAR} is applied. The PSD is annotated in the segmentations of the micrographs corresponding to darker membrane coloring associated with high protein density. These annotated patches are then projected onto the final mesh either through visual comparison or a script. An integer is assigned as metadata to the set of faces corresponding to the PSD. This metadata is passed into `TetGen` which preserves the assigned values. The resulting surface and tetrahedral meshes will contain this additional boundary marking metadata. Softwares such as `FEniCS` can read this metadata and can be thus instructed to model fluxes on some subset of the surface.

We have reworded the sections on boundary marking to help emphasize our intended meaning and context.

"However, in subcellular scenes where the geometry may be tortuous, local receptor clusters can be arbitrarily distributed on the manifold, and the resolution may be insufficient to resolve these features, boundary definition is a non-trivial challenge. In many scenarios, the most biologically accurate boundary definition may be based off of a biologist's understanding of the specimen which transcends the particular dataset. For example, a particular image may not be able to resolve how receptors are distributed but knowledge of relevant immunogold labeling studies may allow the biologist to propose a physiologically meaningful receptor distribution."

page 14. Estimation the Gauss curvature from the angle defect. If you assume that there exists

an underlying smooth surface, the angular defect does not yield a correct estimate of G, see Borrelli, Vincent, Frédéric Cazals, and J-M. Morvan. "On the angular defect of triangulations and the pointwise approximation of curvatures." Computer Aided Geometric Design 20.6 (2003): 319-341.

We thank the reviewer for bring this to our attention. We have added an implementation of Cazals and Pouget's curvature estimator[20, 21]. To prevent future misconceptions related to the angular defect relation to the Gaussian curvature, we have added the following text.

"We note that the curvature values produced by `GAMer 2` are estimates suitable for qualitative comparison and visualization only. The JETS algorithm fits an osculating jet to a local patch using interpolation or approximation. From this fitted jet, the curvature is calculated. Depending on the fineness of the mesh, the jet order, and the details of patch selection, the error in the curvature may vary. On the other hand, Borelli et al. has previously showed that the estimation of the Gaussian curvature using the angle defect, which is used by the MDSB algorithm, is valid only for regular meshes with a vertex valency of 6[22]. The robust calculation of curvature estimates from discrete meshes remains an open problem and the accuracy of many approaches are surveyed by Váša et al. in Ref. [23]."

Experiments: meshes and their . The focus on the improvement of triangle angles is great, but to fully assess the significance of the comparison, the following critical things are missing: - which kind of mesh does IMOD produce? - what is the distribution of angles obtained using state of the art methods such as those provided in CGAL and/or the aforementioned paper by Benes et al?

We thank the reviewer for raising these important points. In Figs. 6, 7, and 11 we show examples of the initial meshes generated by `IMOD` (which implements algorithms adapted from Refs. [24] and [25]) and the distributions of triangular angles.

We have added Fig. 7 which compares the distribution of angles for meshes produced by state-of-the-art methods including `CGAL` 3D Mesh Generation, Hu et al. Remesh, as well as `TetWild` and `VolRoverN`. Where possible we have also generated tetrahedral meshes and computed tetrahedral radius ratios. We find that the surface meshes and tetrahedral meshes produced by `GAMer 2` have comparable mesh quality in comparison to these other methods. In addition to producing quality surface and tetrahedral meshes as compared with other methods, we believe that the surface fairing features of our approach produce a final product which better resembles the underlying biological system by removing high frequency noise which arises from prior workflow steps (e.g., image collection and segmentation).

Experiments: meshes and their topology. Are the meshed you produce watertight? could you report Betti numbers, in order to make sure that the topology of the regions being meshes is correct / preserved?

We thank the reviewer for the excellent feature recommendation. `GAMer 2` has been updated to include the capability to compute Betti numbers. The process is outlined in the section "GAMer 2 Operations Preserve Mesh Topology" and the Betti numbers for all of the meshes used in this paper are reported in Table 2. We make note of two key insights from this topological analysis: 1) Our meshes preserve their topology under `GAMer 2` operations. 2) The Betti numbers of the initial meshes are drastically different from the corrected meshes. Proofing the initial meshes against the images reveals that, in many cases, an error in segmentation has been made.

In some other situations, the meshes produced by `imod2obj` can contain non-manifold features such as edges connected to three faces. Softwares which expect valid surface meshes as input without verification that the input is reasonable are prone to crashing.

Have all data underlying the figures and results presented in the manuscript been provided?

Large-scale datasets should be made available via a public repository as described in the PLOS Computational Biology data availability policy, and numerical data that underlies graphs or summary statistics should be provided in spreadsheet form as supporting information.

Reviewer 1: Yes

Reviewer 2: No: Currently only the source code of GAMer 2 is provided but not the EM images, contour reconstructions and meshes presented in the examples. If they can not be provided due to the ownership, a statement must be included in the Data Availability Statement according to the data availability policy.

We thank the reviewer for bring up this important point on research reproducibility. We have added a supplemental zip file containing `Blender` files with initial and final meshes. The original EM images, segmentation, and contours are the published intellectual property of Wu et al.[26] and are available according to the PNAS data availability policy. These data can be obtained from Wu et al. directly by request. We have included a Data Availability Statement stating this.

Reviewer 3: Yes

Reviewer 4: Yes

References

[1] Christopher T. Lee et al. "An Open Source Mesh Generation Platform for Biophysical Modeling Using Realistic Cellular Geometries". en. In: *bioRxiv* (Sept. 2019), p. 765453. DOI: `10.1101/765453`.

[2] Yixin Hu et al. "Tetrahedral Meshing in the Wild". In: *ACM Trans. Graph.* 37.4 (July 2018), 60:1–60:14. ISSN: 0730-0301. DOI: `10.1145/3197517.3201353`. URL: `http://doi.acm.org/10.1145/3197517.3201353`.

[3] Kaimo Hu et al. "Error-Bounded and Feature Preserving Surface Remeshing with Minimal Angle Improvement". In: *IEEE Transactions on Visualization and Computer Graphics* 23.12 (Dec. 2017), pp. 2560–2573. ISSN: 1077-2626. DOI: `10.1109/TVCG.2016.2632720`.

[4] J. Edwards et al. "VolRoverN: enhancing surface and volumetric reconstruction for realistic dynamical simulation of cellular and subcellular function". In: *Neuroinformatics* 12.2 (Apr. 2014), pp. 277–289.

[5] Pierre Alliez et al. "3D Mesh Generation". In: *CGAL User and Reference Manual*. 5.0. CGAL Editorial Board, 2019. URL: `https://doc.cgal.org/5.0/Manual/packages.html#PkgMesh3`.

[6] M E Rognes et al. "Automating the solution of PDEs on the sphere and other manifolds in FEniCS 1.2". en. In: *Geoscientific Model Development* 6.6 (Dec. 2013), pp. 2099–2119.

[7] Diana C. Resasco et al. "Virtual Cell: Computational Tools for Modeling in Cell Biology". eng. In: *Wiley Interdisciplinary Reviews. Systems Biology and Medicine* 4.2 (2012 Mar-Apr), pp. 129–140. ISSN: 1939-005X. DOI: `10.1002/wsbm.165`.

[8] Rex A. Kerr et al. "Fast Monte Carlo Simulation Methods for Biological Reaction-Diffusion Systems in Solution and on Surfaces". In: *SIAM Journal on Scientific Computing* 30.6 (Jan. 2008), pp. 3126–3149. ISSN: 1064-8275. DOI: `10.1137/070692017`.

[9] Joel R. Stiles and Thomas M. Bartol. "Monte Carlo Methods for Simulating Realistic Synaptic Microphysiology Using MCell". In: *Computational Neuroscience: Realistic Modeling for Experimentalists*. Ed. by Erik De Schutter. 2001, pp. 87–127.

[10] J. R. Stiles et al. "Miniature Endplate Current Rise Times Less than 100 Microseconds from Improved Dual Recordings Can Be Modeled with Passive Acetylcholine Diffusion from a Synaptic Vesicle". eng. In: *Proceedings of the National Academy of Sciences of the United States of America* 93.12 (June 1996), pp. 5747–5752. ISSN: 0027-8424. DOI: `10.1073/pnas.93.12.5747`.

[11] Steven S. Andrews and Dennis Bray. “Stochastic Simulation of Chemical Reactions with Spatial Resolution and Single Molecule Detail”. en. In: *Physical Biology* 1.3 (Aug. 2004), pp. 137–151. ISSN: 1478-3975. DOI: 10.1088/1478-3967/1/3/001.

[12] Iain Hepburn et al. “STEPS: Efficient Simulation of Stochastic Reaction–Diffusion Models in Realistic Morphologies”. In: *BMC Systems Biology* 6.1 (May 2012), p. 36. ISSN: 1752-0509. DOI: 10.1186/1752-0509-6-36.

[13] I. Hepburn, W. Chen, and E. De Schutter. “Accurate Reaction-Diffusion Operator Splitting on Tetrahedral Meshes for Parallel Stochastic Molecular Simulations”. In: *The Journal of Chemical Physics* 145.5 (Aug. 2016), p. 054118. ISSN: 0021-9606. DOI: 10.1063/1.4960034.

[14] Weiliang Chen and Erik De Schutter. “Parallel STEPS: Large Scale Stochastic Spatial Reaction-Diffusion Simulation with High Performance Computers”. English. In: *Frontiers in Neuroinformatics* 11 (2017). ISSN: 1662-5196. DOI: 10.3389/fninf.2017.00013.

[15] Brian Drawert, Stefan Engblom, and Andreas Hellander. “URDME: A Modular Framework for Stochastic Simulation of Reaction-Transport Processes in Complex Geometries”. eng. In: *BMC systems biology* 6 (June 2012), p. 76. ISSN: 1752-0509. DOI: 10.1186/1752-0509-6-76.

[16] Elijah Roberts, John E. Stone, and Zaida Luthey-Schulten. “Lattice Microbes: High-Performance Stochastic Simulation Method for the Reaction-Diffusion Master Equation”. en. In: *Journal of Computational Chemistry* 34.3 (2013), pp. 245–255. ISSN: 1096-987X. DOI: 10.1002/jcc.23130.

[17] Johan Hattne, David Fange, and Johan Elf. “Stochastic Reaction-Diffusion Simulation with MesoRD”. en. In: *Bioinformatics* 21.12 (June 2005), pp. 2923–2924. ISSN: 1367-4803. DOI: 10.1093/bioinformatics/bti431.

[18] Rodrigo F. Oliveira et al. “The Role of Type 4 Phosphodiesterases in Generating Microdomains of cAMP: Large Scale Stochastic Simulations”. en. In: *PLOS ONE* 5.7 (July 2010), e11725. ISSN: 1932-6203. DOI: 10.1371/journal.pone.0011725.

[19] Zeyun Yu et al. “Feature-Preserving Adaptive Mesh Generation for Molecular Shape Modeling and Simulation”. In: *J. Mol. Graph. Model.* 26.8 (June 2008), pp. 1370–1380. ISSN: 10933263. DOI: 10.1016/j.jmgm.2008.01.007.

[20] F. Cazals and M. Pouget. “Estimating Differential Quantities Using Polynomial Fitting of Osculating Jets”. In: *Computer Aided Geometric Design* 22.2 (Feb. 2005), pp. 121–146. ISSN: 0167-8396. DOI: 10.1016/j.cagd.2004.09.004.

[21] Marc Pouget and Frédéric Cazals. “Estimation of Local Differential Properties of Point-Sampled Surfaces”. In: *CGAL User and Reference Manual*. 5.0. CGAL Editorial Board, 2019. URL: https://doc.cgal.org/5.0/Manual/packages.html#PkgJetFitting3.

[22] V. Borrelli, F. Cazals, and J.-M. Morvan. “On the Angular Defect of Triangulations and the Pointwise Approximation of Curvatures”. en. In: *Computer Aided Geometric Design* 20.6 (Sept. 2003), pp. 319–341. ISSN: 01678396. DOI: 10.1016/S0167-8396(03)00077-3.

[23] L. Váša et al. “Mesh Statistics for Robust Curvature Estimation”. en. In: *Computer Graphics Forum* 35.5 (2016), pp. 271–280. ISSN: 1467-8659. DOI: 10.1111/cgf.12982.

[24] H Fuchs, Z M Kedem, and S P Uselton. “Optimal surface reconstruction from planar contours”. In: *Commun. ACM* 20.10 (Oct. 1977), pp. 693–702.

[25] Michael Shantz. “Surface Definition for Branching, Contour-defined Objects”. In: *SIGGRAPH Comput. Graph.* 15.2 (July 1981), pp. 242–270.

[26] Yumei Wu et al. “Contacts Between the Endoplasmic Reticulum and Other Membranes in Neurons”. In: *Proc. Natl. Acad. Sci. USA* 114.24 (June 2017), E4859–E4867. ISSN: 0027-8424. DOI: 10.1073/pnas.1701078114.
