# Supplementary material for: 3D mesh processing using GAMer 2 to enable reaction-diffusion simulations in realistic cellular geometries
Source: PLoS Comput Biol. 2020 Apr 6;16(4):e1007756. doi: 10.1371/journal.pcbi.1007756 (PMC7162555; doi:10.1371/journal.pcbi.1007756)
Supplement: Attachment — Submitted filename: response2.pdf [file pcbi.1007756.s008.pdf]

Response to Reviewers:

We thank the reviewers for their extensive, diligent, and insightful comments. We have responded to specific concerns below and modified the manuscript accordingly; changes in the manuscript are highlighted in red in a separate PDF file attached for review only. The reviewer's comments are given in **bold face** with our response in plain text. Where appropriate, portions of the manuscript that are changed are provided.

Reviewer 1:

I think authors have done a great job, and they certainly have carefully addressed the points I raised. I still have a couple of points that I would like to be clarified:

1 - would be useful to have a screenshot of the application running on blender. Regarding this point, they should also verify whether it runs on the latest version of Blenders which has changed substantially.

`BlendGAMer` runs on all version of `Blender` from v2.79b and onwards. The following text has been added to the second paragraph of §GAMer 2 Development.

"Moreover, the latest `BlendGAMer` release, v2.0.6, supports `Blender` versions 2.79b, and 2.8X."

The following text has been added to the caption of Fig. 5.

"At the time of writing, `BlendGAMer` runs in `Blender` versions ranging from 2.79b and onward."

2 - would be useful to know which kind of computational power is needed to run gamer2. I think it depends of the number of vertices the tool has to process. In particular, does the processing time increase linearly, or exponentially? A basic description of the hardware needed (laptop, desktop, GPU) and a small description of the expected performance would be useful.

We thank the reviewer for highlighting this omission. `GAMer 2` has very limited hardware requirements. The meshes in this work were all conditioned on an older MacBook Air. To emphasize the linear runtime complexity of the algorithms and some directions for future improvement, the following sentences have been added to the discussion:

"The surface mesh conditioning algorithms in `GAMer 2` are local operations which therefore scale linearly with the number of vertices. The hardware requirements to run `GAMer 2` are modest and all meshes shown in this work were processed on a laptop. In the future, `GAMer 2` can be improved to support parallel (shared and/or distributed) processing and conditioning on GPUs. We refer the reader to Ref. [1] for the analysis of runtime complexities of algorithms in `TetGen`."

Reviewer 2:

The manuscript has been greatly improved. I have some comments regarding the Blender plugin and Fig1, but otherwise I am satisfied with this revision.

Fig 1. "GAMer" between subfigure c and d should be "GAMer 2". This pipeline can be described in a more generic way to highlight the software's flexibility, since GAMer 2 doesn't directly couple with either IMOD or FEniCS. It may be more interesting to general readers if the supported input/output formats are given here.

The text "GAMer" in Fig. 1 has been changed to "GAMer 2". Furthermore, the following text has been added to the caption to highlight the generality of `GAMer 2` and the associated algorithms.

"Although this pipeline illustrates an application for images from electron microscopy, `GAMer 2` is a general mesh conditioning library and can be used with meshes regardless of experimental context."

L261 BlenderGAMer section: My main concern with this plugin is that Blender can only

deal with surface mesh. According to my test with the current implementation of GAMer2, while the boundary tags are transferred from the boundary surface mesh to the corresponding triangles in the tetrahedral mesh, they are not propagated to the tetrahedrons. This may be sufficient for FEM simulations, but is less ideal for simulations that require the categorization of subregion tetrahedrons. I suggest the author briefly discuss the possible solution either here or the in discussion, and improve the implementation in the future.

We thank the reviewer for bringing this to our attention. We believe the reviewer may be referring to two possibilities that are addressed as follows:

1. `GAMer 2` supports the marking of regions enclosed by a surface using `TetGen`. The user can specify in `BlendGAMer` region marker values which are used by `TetGen` to mark the enclosed tetrahedra. While this was the intended functionality, in the version of `BlendGAMer` used by the reviewer (v2.0.5 or earlier) we have found a bug where region marker values were not correctly saved when the tetrahedral mesh was retrieved from `TetGen`. This bug has been fixed in PR #46 and will be released with v2.0.6. Volume region markers assigned by the user are now correctly propagated to the tetrahedrons. To clarify this point, the following sentence has been added to the methods:

"Each surface mesh can also be assigned a region marker which is used by `TetGen` to assign marker values for the enclosed tetrahedra."

2. The reviewer may wish to mark the tetrahedra in a subregion with a value of interest. For example, using data from a fluorescence dataset to demarcate the localization of a species in the volume. In this case, the reviewer aptly highlights that `Blender` only deals with surface meshes. While, at this point, `GAMer 2` does not support a graphical approach to marking arbitrary subregions, the assignment of tetrahedral markers can be performed pragmatically through the scripting of libGAMer or PyGAMer. We may develop graphical support for this in future work.

Reviewer 4:

Overview

I am personally quite happy with the revision. From a geometric modeling perspective, the revised version indeed

- **presents in many places a more accurate and specific description of the biophysical problems,**
- **contains a convincing evaluation of meshing algorithms (see one point below, though),**
- **contains a discussion of the difficulties inherent to curvature calculations in the discrete setting,**
- **incorporates a calculation of Betti numbers (see one point below, though).**

There are a number of technical points which need to be polished, though. see details below.

Details

Betti numbers. I am sorry, I did not get your explanation on the modified Edelsbrunner-Delfinado algorithm. on the other hand, if you focus on closed - orientable surfaces, then, by the Thm of classification for such surfaces, one has:

$$\textbf{Euler char} = N_{\textbf{vertices}} - N_{\textbf{edges}} + N_{\textbf{triangles}} = 2 - 2 * genus = \beta_0(=1) - \beta_1 + \beta_2(=1) = 2 - \beta_1.$$

Thus, computing the Betti numbers is trivial from the Euler char, which is trivial from the triangle mesh.

We apologize for the confusion with this section. We acknowledge the approach described by the reviewer which assumes that the mesh is both closed and orientable. For our implementation, we do not assume that the input mesh is closed and orientable. The algorithm described can compute the Betti numbers of a mesh so long as there are no edges participating in three or more faces. For other meshes, including for example a lone triangle with no face (3 connected edges), the first three Betti numbers are correctly computed and reported. The relevant text has been modified for clarity and reproduced here:

"We simplify the problem at hand by restricting our calculation to apply for the set of topologically manifold surface meshes with or without boundaries. If a mesh is both orientable (i.e., a consistent normal orientation can be assigned for all faces such that no neighboring faces have opposing normals) and all edges belong to two faces, we say that the mesh is a closed manifold. After iterating through a filtration in the manner described by Delfinado-Edelsbrunner, if we find that a mesh is a closed manifold then we increment the first and second Betti numbers (β_1, β_2) by one. If the mesh is not a closed manifold, than nothing is done and the algorithm terminates and reports the first three Betti numbers. At any point iterating through the filtration, if an edge is found to participate in three or more faces, the mesh is not topologically manifold and only the zeroth Betti number is reported."

I also note that lines with large β_1 means that the surface has numerous tiny handles. These should be filtered out using topological persistence – see the Gudhi library.

We thank the reviewer for this helpful suggestion. The capability of topological persistence to filter these holes has been noted and the following sentence has been added to the text.

"As an alternative to manual curation, other approaches such as persistent homology can be used to filter out defects [2]."

Curvatures: jet fitting versus MDSB. for Jet fitting, you need to specify the degree of the jet and the number of neighbors. jet fitting without these parameters does not make sense.

We apologize for this omission. We are fitting a second degree jet to six neighbors. The following text has been added to the figure caption.

"For the JETS estimate a second degree jet was fit to six neighboring points for all meshes."

Curvatures, sign. about "We have adopted the sign convention where negative curvature values refer to convex regions." you should be more precise: what the the relationship between convexity and the local nature of the point (elliptic, hyperbolic, parabolic) !???

We have added a table describing the local nature of the point according to our sign convention to the Supplementary Information. A reference to this table has been added in the main text where we describe the sign convention.

TetGen and constrained Delaunay. the main text should mention the fact that TetGen uses a constrained Delaunay triangulation.

This fact has been added to the main text in the methods section.

"After boundaries are marked, stacks of surface meshes corresponding to different domains can be grouped and passed from `GAMer 2` into `TetGen`, which generates a volume mesh by constrained Delaunay tetrahedralization[1]."

Contenders and reproducibility of results, Fig. 7. one reads in the answers that "We found that for some codes such as VolRoverN, Hu et al. Remesh, and CGAL Mesh 3, many input meshes would produce segmentation fault."

Since CGAL is supposed to be robust, we face two alternatives here: either the program has not been compiled and/or user properly. (e.g., certain aggressive optimization options may jeopardize the numerics); or there is a bug.

To clarify the situation, the supporting material should contain the protocol used, and provide

the files where the tests failed.

We thank the reviewer for this excellent suggestion. We have added to the Supporting Information the protocols used with each software for mesh generation. Furthermore the surface meshes input to each software are also provided.

With respect to software robustness, the downloaded versions of `VolRoverN` and Hu et al., Remesh are consistently problematic. `CGAL` on the other hand is indeed usually robust. As the reviewer mentions, only in cases where the user has inadvertently provided an erroneous input or specified overly aggressive optimization settings does the code misbehave. The example scripts provided with `CGAL` are helpful but simple workflows. We do not believe these examples were intended for robust use in production and certain checks for mesh integrity are not performed. We believe that the construction of a mesh generation code, using `CGAL`, which is robust to these issues is possible but beyond the scope of this work.

Have all data underlying the figures and results presented in the manuscript been provided?

Large-scale datasets should be made available via a public repository as described in the PLOS Computational Biology data availability policy, and numerical data that underlies graphs or summary statistics should be provided in spreadsheet form as supporting information.

Reviewer 1: Yes

Reviewer 2: Yes

Reviewer 4: Yes

References

[1] Hang Si. "TetGen, a Delaunay-Based Quality Tetrahedral Mesh Generator". In: *ACM Trans. Math. Softw.* 41.2 (Feb. 2015), pp. 1–36. ISSN: 00983500. DOI: 10.1145/2629697.

[2] Nina Otter et al. "A Roadmap for the Computation of Persistent Homology". en. In: *EPJ Data Science* 6.1 (Dec. 2017), pp. 1–38. ISSN: 2193-1127. DOI: 10.1140/epjds/s13688-017-0109-5.
